# Phosphorylation of Histone H2A at Serine 95 Is Essential for Flowering Time and Development in *Arabidopsis*

**DOI:** 10.3389/fpls.2021.761008

**Published:** 2021-11-23

**Authors:** Tongtong Huang, Heng Zhang, Yiming Zhou, Yanhua Su, Han Zheng, Yong Ding

**Affiliations:** Ministry of Education Key Laboratory for Membraneless Organelles and Cellular Dynamics, Hefei National Laboratory for Physical Sciences at the Microscale, Division of Molecular Cell Biophysics, Chinese Academy of Sciences (CAS) Center for Excellence in Molecular Plant Sciences, School of Life Sciences, Division of Life Sciences and Medicine, Biomedical Sciences and Health Laboratory of Anhui Province, University of Science and Technology of China, Hefei, China

**Keywords:** histone phosphorylation, flowering time, *GI*, MLK family, phosphorylation of histone H2A at serine 95

## Abstract

Phosphorylation of H2A at serine 95 (H2AS95ph) mediated by MLK4 promotes flowering and H2A.Z deposition. However, little is known about MLK1, MLK2, and MLK3 during the flowering time. Here, we systemically analyze the functions of MLK family in flowering time and development. Mutation in *MLK3*, but not *MLK1* and *MLK2*, displayed late-flowering phenotype. Loss of *MLK3* function enhanced the late-flowering phenotype of *mlk4* mutant, but not reinforced the late-flowering phenotype of *mlk1 mlk2* double mutants. MLK3 displayed the kinase activity to histone H2AS95ph *in vitro*. The global H2AS95ph levels were reduced in *mlk3 mlk4*, but not in *mlk3* and *mlk4* single mutant and *mlk1 mlk2* double mutant, and the H2AS95ph levels in *mlk1 mlk3 mlk4* and *mlk2 mlk3 mlk4* were similar to those in *mlk3 mlk4* double mutant. MLK3 interacted with CCA1, which binds to the promoter of *GI*. Correspondingly, the transcription levels and H2AS95ph levels of *GI* were reduced in *mlk3* and *mlk4* single mutant, and greatly decreased in *mlk3 mlk4* double mutant, but not further attenuated in *mlk1 mlk3 mlk4* and *mlk2 mlk3 mlk4* triple mutant. Together, our results suggested that H2AS95ph deposition mediated by MLK3 and MLK4 is essential for flowering time in *Arabidopsis*.

## Introduction

The N-terminal tails of the core histones in nucleosomes are subjected to posttranslational modifications such as acetylation, methylation, phosphorylation, ubiquitination, glycosylation, ADP-ribosylation, carbonylation, and sumoylation. The phosphorylation generally occurs at the serine10, serine28, threonine3, and threonine11 at the N-terminus of H3, and these phosphorylation sites are primarily linked to chromosome condensation/segregation, transcription activation, and apoptosis (Wang et al., 2011; Pirrotta, 2015). Phosphorylation of histone H3 at threonine 3 (H3T3ph) by Haspin during mitosis is accompanied by chromatin condensation, and it provides a chromatin binding site for the chromosomal passenger complex at centromeres to regulate chromosome segregation (Kelly et al., 2010; Wang et al., 2011; Pirrotta, 2015). MSK1/2-mediated phosphorylation of H3 serine 10 and serine 28 is coupled with transcription activation through antagonizing polycomb silencing (Soloaga et al., 2003; Lau and Cheung, 2011). Protein-kinase-C-related kinase 1 (PRK1) phosphorylates histone H3 at threonine 11 (H3T11ph) upon ligand-dependent recruitment to androgen receptor target genes (Metzger et al., 2008). In contrast to phosphorylation of H3, H2A phosphorylation is mostly well known in DNA damage. In yeast, H2A phosphorylation at serine 129 is essential for the repair of DNA double-stranded breaks by recruiting the INO80 complex (Van Attikum et al., 2004). In addition to DNA damage, H2A phosphorylation at threonine 133 is associated with centromere function and maintenance during meiosis in maize (Dong and Han, 2012). Phosphorylation of H2A at serine 95 (H2AS95ph) is associated with transcription activation in *Arabidopsis* (Su et al., 2017).

The histone phosphorylation is linked with histone methylation, histone acetylation, and histone variants. Phosphorylation of H3T11 by PRK1 accelerates demethylation by the Jumonji C (JmjC)-domain-containing protein JMJD2C (Metzger et al., 2008). Phosphorylation of histone H3 at threonine 6 (H3T6ph) by protein kinase C beta I prevents LSD1 from demethylating H3K4 during AR-dependent gene activation (Metzger et al., 2010). In *Chlamydomonas*, phosphorylation of histone H3 at threonine 3 (H3T3ph) by MUT9p is responsible for repression of transgenes and transposons, and the loss of *MUT9p* function resulted in a reduction of H3T3ph levels with the induction of H3K4me2 and H3K4me3 (Casas-Mollano et al., 2008). Phosphorylation of H3 serine 10 is tightly linked to acetylation of H3K9 and H3K14 (Winter et al., 2008). In addition to histone methylation and acetylation, phosphorylation of histone H2A at serine 95 (H2AS95ph) promotes SWR1 complex recruitment and H2A.Z enrichment (Su et al., 2017).

Flowering at an appropriate time is crucial for plant life cycles. The flowering time is initially in response to endogenous controls and environmental cues (Boss et al., 2004; Amasino and Michaels, 2010). The two common environmental factors are day length (photoperiod) and temperature (Michaels et al., 2005; Amasino and Michaels, 2010). Plant perceives the seasonal changes in day length to regulate flowering time. Photoperiod is the primary environmental signal regulating flowering time in summer-annual *Arabidopsis* with flowering rapidly under long days (LD) and slowly under short days (SD). *CONSTANS (CO)* encodes a B-box zinc-finger protein that activates the expression of *FT* and acts as a suppressor of the overexpression of *CO 1* (*SOC1*) (Putterill et al., 1995; Samach et al., 2000; Song et al., 2015). *CO* primarily accelerates flowering under LD; and loss of *CO* function resulted in late flowering under LD, but not SD (Putterill et al., 1995; Song et al., 2015). *CO* transcription levels were regulated by *flavin-binding, Kelch repeat, F-box 1* (*FKF1*), and *Gigantea* (*GI*) (Sawa et al., 2007). FKF1 and GI form a complex in the late afternoon and promote the *CO* transcription; thus, the transcript levels of *CO* reach the peak at the end of a long day (Sawa et al., 2007; Song et al., 2015). *Arabidopsis* flowering time in photoperiod pathway also requires components involved in circadian rhythms. *Circadian clock associated 1* (*CCA1*) and *late elongated hypocotyl* (*LHY*) encode MYB-like transcription factors (Wang and Tobin, 1998; Mizoguchi et al., 2002). CCA1 and LHY formed a central oscillator, and the loss of *CCA1* and *LHY* function displayed dramatically earlier phases of expression of *GI* (Mizoguchi et al., 2002, 2005).

Casein kinase I, a serine/threonine protein kinase, is conserved from yeast to mammalian cells, and plants (Cruciat, 2014). Casein kinase I (CKI) members play a critical role in Wnt signaling, circadian rhythms, and multiple cellular processes (Lin et al., 2002; Cruciat, 2014; Su et al., 2017). In *Arabidopsis*, CKI is involved in root development, ethylene synthesis, flowering time, and circadian clock (Liu et al., 2003; Ogiso et al., 2010; Tan and Xue, 2014; Uehara et al., 2019). Chlamydomonas MUT9p is closely related to casein kinase I and possesses kinase activity to histone H2A and H3 (Casas-Mollano et al., 2008). Mapping of phosphorylation sites show that MUT9p prefers to phosphorylate histone H3 at threonine 3 (Casas-Mollano et al., 2008). *Arabidopsis* genome encodes four MUT9p-like kinase/photoregulatory protein kinases/Arabidopsis EL1-like kinases, herein referred to as the MLKs/AELs, namely, MLK1, MLK2, MLK3, and MLK4 (Casas-Mollano et al., 2008; Huang et al., 2016). *MLK1* and *MLK2* are involved in osmotic stress and hypocotyl elongation (Wang et al., 2015; Zheng and Ding, 2018; Zheng et al., 2018). MLK4 was firstly identified from the evening clock complex (Huang et al., 2016). The following study showed that MLK4 phosphorylated H2AS95ph *in vitro* and *in vivo* (Su et al., 2017). MLK4 interacts with CCA1, and loss of *MLK4* functions resulted in late flowering and reduction of H2AS95ph and H2A.Z level at *GI* (Su et al., 2017). A recent study showed that MLK4 deposited H3T3ph at *FLC/MAF* (Wang et al., 2021). Phosphoproteomics showed that MLKs phosphorylated proteins in circadian clock, multiple metabolic, and signaling pathways in *Arabidopsis* (Wilson et al., 2021). MLKs/AELs are also involved in abscisic acid signaling pathway (Chen et al., 2018), suggesting that the functions of MLKs are complicated and need further study.

The amino acid of H2A is not conserved as that of H3 in different species (Kawashima et al., 2015). The sequence diversity of H2A determines the modification sites complexity from yeast to mammalian cells, and plants. Compared with the phosphorylation of H3, less is known about phosphorylation at H2A. Most importantly, the functions and phosphorylation sites of MLKs are elusive. Here, we comprehensively revealed the phosphorylation activity and flowering time of *MLK*s. Loss of *MLK3* function exhibited late-flowering phenotype under LD and reinforced the late-flowering phenotype of *mlk4*. Mutations of *MLK1 or MLK2* in *mlk3 mlk4* double mutant do not enhance the late-flowering phonotype of *mlk3 mlk4* plants. MLK3 phosphorylated histone H2AS95ph *in vitro* and *in vivo*. The global levels of H2AS95ph were reduced in *mlk3 mlk4* double mutant, but were not further reduced in *mlk1 mlk3 mlk4* or *mlk2 mlk3 mlk4* triple mutants. In addition, MLK3 interacts with CCA1 and binds to the *GI* promoter. The transcription levels and H2AS95ph levels at *GI* were reduced in *mlk3 mlk4* double mutants, and in *mlk1 mlk3 mlk4* and *mlk2 mlk3 mlk4* triple mutants. Together, our results showed that MLKs prefer to phosphorylate H2AS95ph *in vitro* and *in vivo*, and *MLK3* and *MLK4* are the major components in flowering time and histone 2A phosphorylation at serine 95.

## Results

### *MLK3* Is Involved in Photoperiod Flowering Pathway

**FIGURE 1:**
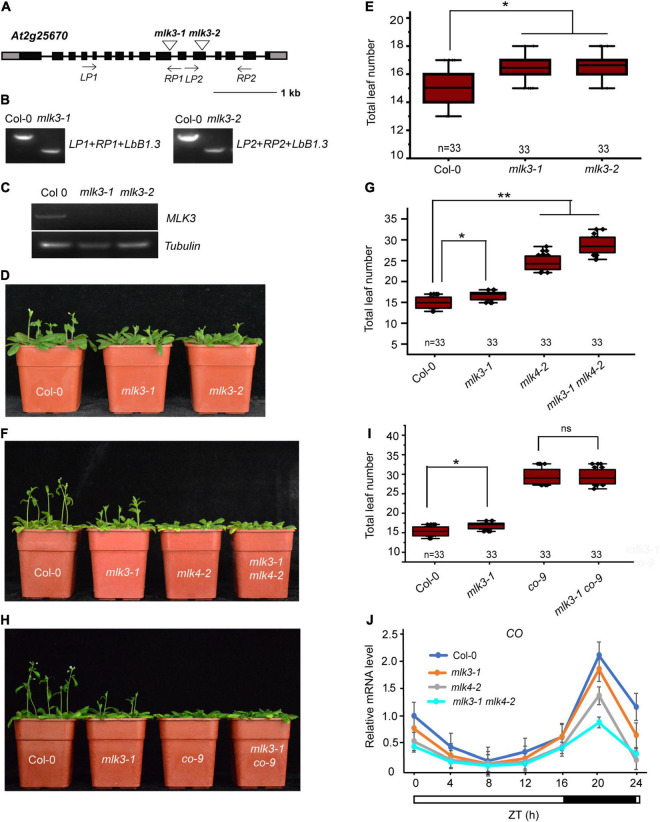
*MLK3* is involved in photoperiod flowering time pathway. **(A)** Gene structure of *MLK3*, indicating exons (boxes), introns (lines), and T-DNA insertions (triangles). The primers used for genotype analysis are marked with arrows. **(B)** Genotypic analysis of the *mlk3* mutants. The genotype was analyzed with the left genomic primer (LP), right genomic primer (RP), and the vector primer (LBb1.3). The positions of LP1, RP1, LP2, and RP2 are indicated in panel **(A)**. **(C)** The full-length *MLK3* transcript in the *mlk3* mutants was examined by RT-PCR. **(D)** The representative phenotype of Col-0 and *mlk3* mutants under a LD photoperiod. **(E)** The total leaf number of Col-0 and *mlk3* mutants under a LD photoperiod. Flowering time was assessed by counting the number of rosette leaves and cauline leaves at bolting under LD. Values shown are mean ± SD of total leaves; 33 plants were scored for each line. Asterisks indicate *P* < 0.05 by *t*-test, ns indicates no significance. **(F)** The representative phenotype of Col-0, *mlk3-1*, *mlk4-2*, and *mlk3-1 mlk4-2* plants under a LD photoperiod. **(G)** The total leaf number of Col-0, *mlk3-1*, *mlk4-2*, and *mlk3-1 mlk4-2* mutants under a LD photoperiod. Values shown are mean ± SD of total leaves; 33 plants were scored for each line. Asterisks indicate *P* < 0.05 by *t*-test, ns indicates no significance. **(H)** The representative phenotype of Col-0, *mlk3-1*, *co-9*, and *mlk3-1 co-9* plants under a LD photoperiod. **(I)** The total leaf number of Col-0, *mlk3-1*, *co-9*, and *mlk3-1 co-9* mutants under a LD photoperiod. Values shown are mean ± SD of total leaves; 33 plants were scored for each line. Asterisks indicate *P* < 0.05 and ns indicates no significance by *t*-test. **(J)** The transcript levels of *CO* were examined in Col-0, *mlk3-1*, *mlk4-2*, and *mlk3-1 mlk4-2* mutants. The white bar indicates the light periods, and the black bar indicates the dark period. ZT, Zeitgeber time. Experiments were repeated at least three times, and the representative experiments shown indicate the mean ± SE, *n* = 3 replicates.

Phylogenetic analysis showed MLK3 is closely related to MLK1, MLK2, and MLK4 (Supplementary Figure 1). To investigate the function of *MLK3*, we isolated the two T-DNA insertion mutants. Genotyping showed that the *mlk3-1* and *mlk3-2* alleles, each contain a T-DNA in the exon10 and exon12, respectively (Figures 1A,B). RT-PCR analyses revealed that *mlk3-1* and *mlk3-2* are null alleles (Figure 1C). The primary leaf number at bolting of *mlk3-1* and *mlk3-2* is more than that of the wild type (Supplementary Table 1). *mlk3-1* and *mlk3-2* exhibited late flowering-time phenotype under long day (LD) photoperiod, but not in short day (SD) photoperiod (Figures 1D,E and Supplementary Table 1), suggesting that *MLK3* is involved in photoperiod pathway.

To investigate the redundant functions of *MLK3* and *MLK4*, we crossed *mlk3-1* into *mlk4-2* and generated the *mlk3-1 mlk4-2* double mutant. The flowering time of *mlk3-1 mlk4-2* double mutant is later than those of *mlk3-1* and *mlk4-2* in LD, but not in SD (Figures 1F,G, and Supplementary Table 1). *mlk3-2 mlk4-3* double mutant further supported that the functions of *MLK3* and *MLK4* in flowering time (Supplementary Table 1). These results suggested that *MLK3* and *MLK4* have unequal redundancy in flowering time.

To confirm this, we crossed the *mlk3-1* into *co-9* and generated *mlk3-1 co-9* double mutant. The flowering time of *mlk3-1 co-9* is similar to that of *co-9* (Figures 1H,I and Supplementary Table 1), suggesting that *MLK3* is involved in *CO*-dependent flowering pathway. The transcripts of *CO* were examined, and the transcription levels of *CO* were reduced in *mlk3* mutants, and further reduced in *mlk3 mlk4* mutants (Figure 1J and Supplementary Figure 2A), suggesting that *CO* is downstream of *MLK3* and *MLK4*. We then investigated transcripts of *FT* and found that the transcript levels of *FT* were reduced in *mlk3*, *mlk4*, and *mlk3 mlk4* plants, which are positively correlated with transcript levels of *CO* (Supplementary Figure 2B). We then investigated the transcripts of *MLK3* and found that *MLK3* exhibits a circadian rhythm dependent expression pattern with a peak at the end of the 16-h light period, which is similar to the expression patterns of *CO* and *FT* (Supplementary Figures 2A–C). However, the transcript levels of *FLC* did not show an obvious difference in *mlk3*, *mlk4* single mutants, and *mlk3 mlk4* double mutants (Supplementary Figure 2D).

### *MLK1* and *MLK2* Are Involved in Photoperiod Flowering Time

**FIGURE 2:**
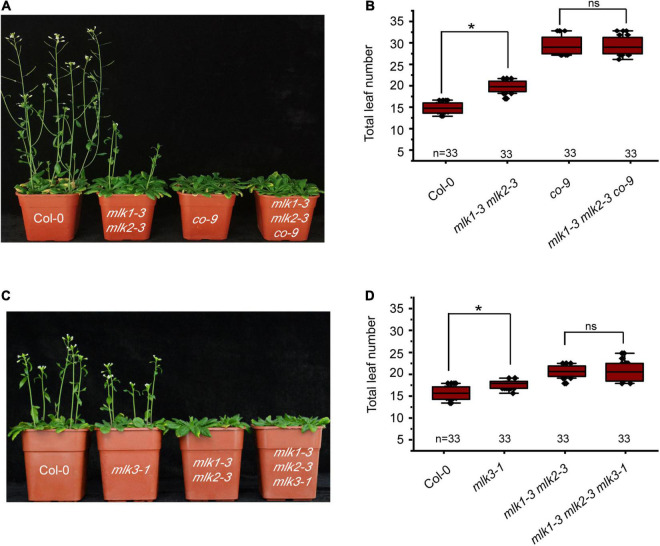
Loss of *MLK1* and *MLK2* function resulted in late-flowering phenotype. **(A)** The representative phenotype of Col-0, *mlk1-3 mlk2-3*, *co-9*, and *mlk1-3 mlk2-3 co-9* plants under a LD photoperiod. **(B)** The total leaf number of Col-0, *mlk1-3 mlk2-3*, *co-9*, and *mlk1-3 mlk2-3 co-9* mutants under a LD photoperiod. Values shown are mean ± SD of total leaves; 33 plants were scored for each line. Asterisks indicate *P* < 0.05 by *t*-test, ns indicates no significance. **(C)** The representative phenotype of Col-0, *mlk1-3 mlk2-3*, *mlk3-1*, and *mlk1-3 mlk2-3 mlk3-1* plants under a LD photoperiod. **(D)** The total leaf number of Col-0, *mlk1 mlk2*, *mlk3*, and *mlk1 mlk2 mlk3* mutants under a LD photoperiod. Values shown are mean ± SD of total leaves; 33 plants were scored for each line. Asterisks indicate *P* < 0.05 and ns indicates no significance by *t*-test.

Mutation in *MLK1* or *MLK2* did not show any flowering-time phenotype, and *mlk1 mlk2* double mutant displayed the late flowering under LD, but not SD (Zheng et al., 2018; Supplementary Figures 3A,B and Supplementary Table 1). We examined whether *MLK1/2* is involved in the photoperiod pathway. We crossed *mlk1-3 mlk2-3* into *co-9* and found that the flowering time of *mlk1-3 mlk2-3 co-9* triple mutant is similar to that of *co-9* (Figures 2A,B, Supplementary Figure 3C, and Supplementary Table 1), suggesting that *MLK/2* is involved in the photoperiod pathway. These results were confirmed with reduced *CO* transcription level in *mlk1-3 mlk2-3* double mutant (Supplementary Figure 3D).

To investigate the relationship between *MLK3* with *MLK1* and *MLK2*, we crossed *mlk3-1* into *mlk1-3 mlk2-3* double mutant and generated *mlk1-3 mlk2-3 mlk3-1* triple mutant. The flowering time of *mlk1-3 mlk2-3 mlk3-1* triple mutant is similar to that in *mlk1-3 mlk2-3* double mutant in LD and SD (Figures 2C,D and Supplementary Table 1). The transcript levels support these results that the transcription levels of *CO* in *mlk1 mlk2* double mutant are similar to those in *mlk1 mlk2 mlk3* triple mutant (Supplementary Figure 3D).

### *MLK4* Participates in Flowering Time and Plant Development

**FIGURE 3:**
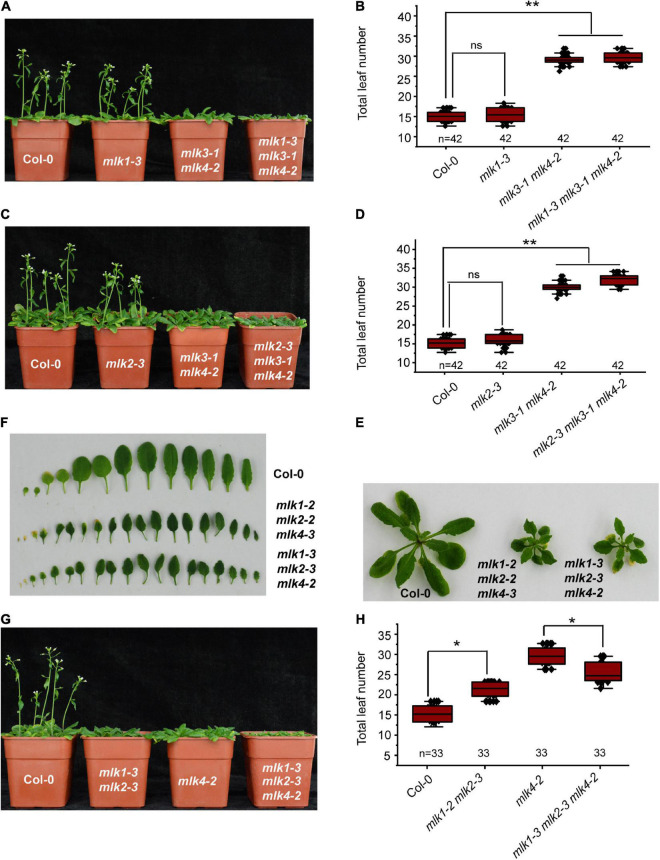
Loss of *MLK1*, *MLK2*, and *MLK4* function resulted in growth defects. **(A)** The representative phenotype of Col-0, *mlk1-3*, *mlk3-1 mlk4-2*, and *mlk1-3 mlk3-1 mlk4-2* plants under a LD photoperiod. **(B)** The total leaf number of Col-0, *mlk1-3*, *mlk3-1 mlk4-2*, and *mlk1-3 mlk3-1 mlk4-2* mutants under a LD photoperiod. Values shown are mean ± SD of total leaves; 33 plants were scored for each line. Asterisks indicate *P* < 0.01 and ns indicates no significance by *t*-test. **(C)** The representative phenotype of Col-0, *mlk2-3*, *mlk3-1 mlk4-2*, and *mlk2-3 mlk3-1 mlk4-2* plants under a LD photoperiod. **(D)** The total leaf number of Col-0, *mlk2-3*, *mlk3-1 mlk4-2*, and *mlk2-3 mlk3-1 mlk4-2* mutants under a LD photoperiod. Values shown are mean ± SD of total leaves; 33 plants were scored for each line. Asterisks indicate *P* < 0.01 and ns indicates no significance by *t*-test. **(E)** The 3-week old *mlk1-3 mlk2-3 mlk4-2* triple mutants exhibit the dwarf phenotype. **(F)** The representative leaves from 3-week old *mlk1-3 mlk2-3 mlk4-2* plants are shown. **(G)** The representative phenotype of Col-0, *mlk1-3 mlk2-3*, *mlk4-2*, and *mlk1-3 mlk2-3 mlk4-2* plants under a LD photoperiod. **(H)** The total leaf number of Col-0, *mlk1-3 mlk2-3*, *mlk4-2*, and *mlk1-3 mlk2-3 mlk4-2* mutants under a LD photoperiod. Values shown are mean ± SD of total leaves; 33 plants were scored for each line. Asterisks indicate *P* < 0.05 by *t*-test.

We next individually examined the *MLK1* and *MLK2* in contribution to flowering time in *mlk3 mlk4* double mutant. We introduced *mlk1-3* and *mlk2-3* into *mlk3-1 mlk4-2*, and generated *mlk1-3 mlk3-1 mlk4-2* and *mlk2-3 mlk3-1 mlk4-2* triple mutant, respectively. The flowering time of *mlk1-3 mlk3-1 mlk4-2* and *mlk2-3 mlk3-1 mlk4-2* is similar to that of *mlk3-1 mlk4-2* under LD (Figures 3A–D and Supplementary Table 1). These observations suggested that mutations of *MLK1* or *MLK2* in *mlk3 mlk4* double mutant failed to reinforce the late-flowering phenotype of *mlk3 mlk4* under LD. Collecting the flowering-time of *mlk3*, *mlk4, mlk3 mlk4, mlk1 mlk3 mlk4*, and *mlk2 mlk3 mlk4* mutants (Figures 1F,G, 2C,D, 3A–D), we concluded that *MLK4* played critical roles in flowering time in MLK family.

The functions of *MLK1/2* and *MLK4* were then investigated. The *mlk1-3 mlk2-3* displayed semidwarf phenotype, whereas *mlk1-3 mlk2-3 mlk4-2* exhibited severe dwarf phenotype with short petiole, sharp, and curly leaf (Figures 3E,F and Supplementary Figure 3E); suggesting that *MLK4* is involved in plant development with *MLK1/2*. The flowering-time of *mlk1-3 mlk2-3 mlk4-2* is early than that of *mlk4-2* under LD (Figures 3G,H). One possibility is due to strong growth defects in *mlk1-3 mlk2-3 mlk4-2* (Figures 3E,F and Supplementary Figure 3E).

### The Global Changes of H2AS95ph in *mlk*s Plants

**FIGURE 4:**
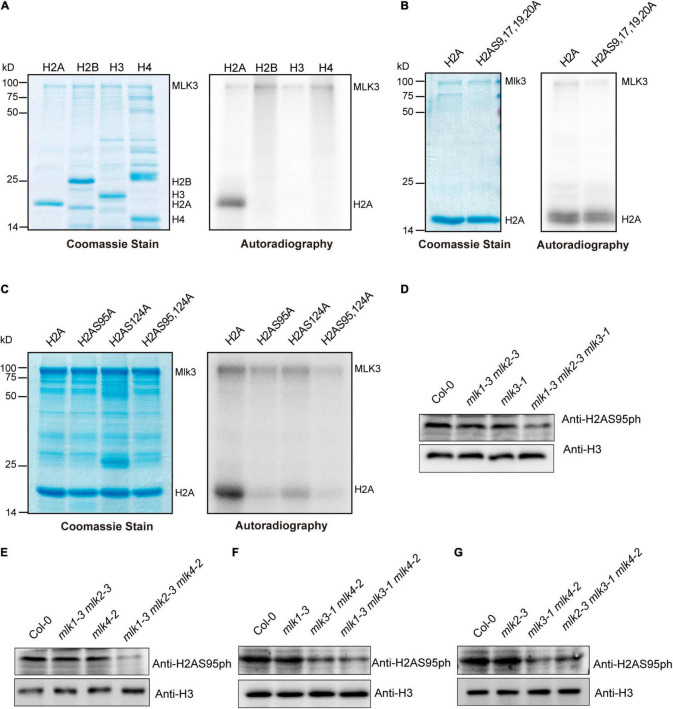
The global changes of H2AS95ph in *mlk*s plants. **(A)** MLK3 phosphorylated H2A. The ability of MLK3 to phosphorylate H2A, H2B, H3, and H4 was assessed. The MLK3, H2A, H2B, H3, and H4 were marked. **(B,C)** The activity and specificity of the MLK3 kinase were assessed using different substrates. Wild-type H2A and H2A containing serine-to-alanine substitutions were used as various residues. **(D–G)** The global levels of H2AS95 phosphorylation were examined in *mlk1*, *mlk2*, *mlk3*, and *mlk4* single mutants, *mlk1 mlk2* and *mlk3 mlk4* double mutants, and *mlk1 mlk2 mlk3, mlk1 mlk2 mlk4, mlk1 mlk3 mlk4*, and *mlk2 mlk3 mlk4* triple mutants. H3 was used as internal control.

We investigated the MLK3 phosphorylation activity and found that MLK3 exhibited high activity to histone H2A (Figure 4A). MLK3 prefer to phosphorylate the serine of H2A and the six serines of H2A are located at amino acid positions 9, 17, 19, 20, 95, and 124. The H2A histones containing serine-to-alanine substitutions at 9, 17, 19, and 20 were strongly phosphorylated, but this phosphorylation activity was impaired when serine 95 and serine 124 were substituted with alanine (Figures 4B,C). Further study showed that serine 95 substituted by alanine primarily abolished the MLK3 activity (Figure 4C). Similar results were observed in MLK1/2 phosphorylation activity assays (Supplementary Figures 4A–F).

The global levels of H2AS95ph were examined and no obvious changes were observed in *mlk1, mlk2, mlk3*, and *mlk4* single mutants and *mlk1 mlk2* double mutants (Figures 4D,E). However, mutations in *MLK3* or *MLK4* in *mlk1 mlk2* double mutant reduced global H2AS95ph levels (Figures 4D,E), suggesting that MLK3 and MLK4 are crucial in H2AS95ph deposition. These results confirmed that reduction of H2AS95ph levels in *mlk3 mlk4* double mutants was observed (Figures 4F,G). In addition, H2AS95ph levels in *mlk1 mlk3 mlk4* and *mlk2 mlk3 mlk4* triple mutants are similar to those of *mlk3 mlk4* double mutants (Figures 4F,G). These observations indicated that the downregulated H2AS95ph levels were correlated with late-flowering time phenotype in *mlk3 mlk4*, *mlk1 mlk3 mlk4*, and *mlk2 mlk3 mlk4* plants. In addition to flowering time, *MLK1* and *MLK2* were also involved in plant development and osmotic stress resistance (Wang et al., 2015; Zheng et al., 2018), suggesting that *MLK1* and *MLK2* might function independently with *MLK3* and *MLK4*.

Since MLK1, MLK2, and MLK4 also functioned as kinases to histone H3 at threonine 3 (Wang et al., 2015, 2021), we examined the H3T3ph levels. No obvious difference of H3T3ph levels were observed in wild type, *mlk1 mlk2 mlk3*, *mlk1 mlk2 mlk4*, *mlk1 mlk3 mlk4*, and *mlk2 mlk3 mlk4* triple mutants (Supplementary Figure 5A). These results suggested that MLKs are predominately involved in H2AS95 phosphorylation, but not H3T3 phosphorylation.

### MLK3 Interacts With CCA1 *in vitro* and *in vivo*

**FIGURE 5:**
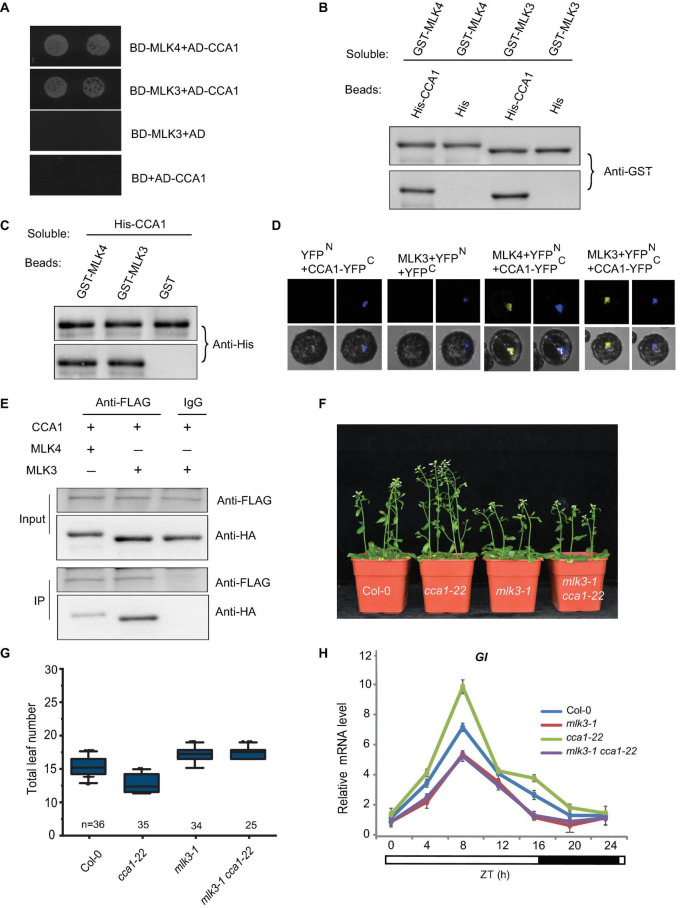
MLK3 interacts with CCA1 *in vitro* and *in vivo*. **(A)** A yeast two-hybrid assay revealed an interaction between MLK3 and CCA1. The growth of two dilutions (2 × 10^–2^ and 2 × 10^–3^) of the yeast culture on SD medium lacking Trp, Leu, His, and adenine is shown. **(B)** Beads containing a His-fused CCA1 were assayed for their ability to bind to soluble GST-fused MLK3. The input and bound proteins were detected with an antibody to GST (anti-GST). MLK4 was used as positive control, and His alone was used as negative control. **(C)** Beads containing a GST-fused MLK3 were assessed for their ability to bind to soluble His-fused CCA1 and detected with antibody to His (anti-His). **(D)** Either MLK3 fused to the N terminus of YFP or the N terminus of YFP alone was tested for their ability to bind to the C terminus of YFP fused to C terminus of YFP fused to CCA1. Yellow fluorescence and a bright-field image were recorded and the resulting images were merged. Twenty-five cells were examined for each transformation. Bar = 10 μm. **(E)** Co-immunoprecipitation of MLK3 and CCA1. FLAG-CCA1 and HA-MLK3 were cotransformed into *Arabidopsis* protoplasts, and the expressed proteins were immunoprecipitated using an anti-FLAG antibody, and detected with anti-Flag and anti-HA. In panels **(A–E)**, experiments were repeated at least three times, and representative experiments are shown. **(F)** The representative phenotype of Col-0, *mlk3-1*, *cca1-22*, and *mlk3-1cca1-22* plants under a LD photoperiod. **(G)** The total leaf number of Col-0, *mlk3-1*, *cca1-22*, and *mlk3-1 cca1-22* plants under a LD photoperiod. Values shown are mean ± SD of total leaves; At least 25 plants were scored for each line. Asterisks indicate *P* < 0.05 and ns indicates no significance by *t*-test. **(H)** The transcript levels of *GI* were examined in Col-0, *mlk3-1*, *cca1-22*, and *mlk3-1 cca1-22* mutants. The white bar indicates the light periods, and the black bar indicates the dark period. ZT, Zeitgeber time. Experiments were repeated at least three times, and the representative experiments shown indicate the mean ± SE, *n* = 3 replicates.

Since MLK3 is involved in photoperiod pathway, we tested whether MLK3 interacts with any of the factors in the circadian rhythm pathway. A yeast two-hybrid result showed that MLK3 directly interacts with CCA1, but not with the controls (Figure 5A). These results were confirmed with pull-down and BiFC. Beads containing His fused with CCA1 (His-CCA1), but not His, bound to soluble GST fused with MLK3 (GST-MLK3) (Figure 5B). Reciprocally, beads containing GST-MLK3 were bound to soluble His-CCA1 (Figure 5C). These results were validated by BiFC, and reconstituted YFP fluorescence was observed in the nucleus with coexpressing MLK3 fused to the YFP N-terminus (MLK3-YFP^*N*^) and CCA1 fused to the YFP C-terminus (CCA1-YFP^*C*^) (Figure 5D). The co-immunoprecipitation (Co-IP) supported our finding that MLK3 directly binds to CCA1. HA fused with MLK3 (HA-MLK3) and FLAG fused with CCA1 (FLAG-CCA1) were coexpressed in *Arabidopsis* protoplasts and immunoprecipitated with anti-FLAG antibody. MLK3, but not control, could be coimmunoprecipitated with CCA1 (Figure 5E).

The *mlk3 cca1* double mutant was then generated by crossing *mlk3-1* into *cca1-22*. Mutations in *MLK3* reversed the early-flowering time of *cca1-22*, and the flowering time of *mlk3-1 cca1-22* is similar to *mlk3-1* plants (Figures 5F,G). Since CCA1 could bind to the promoter of *GI* (Su et al., 2017), we then investigated the transcripts of *GI* and found transcription levels of *GI* in *mlk3-1 cca1-22* are similar to those in *mlk3-1* plants (Figure 5H).

### MLKs Are Involved in H2AS95ph Deposition at *GI*

**FIGURE 6:**
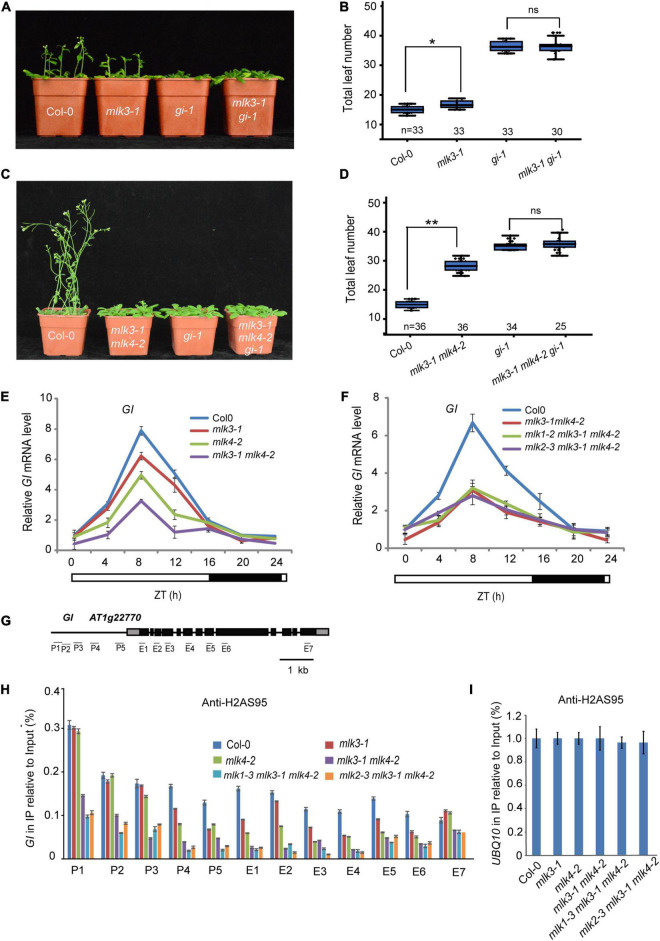
MLK3 and MLK4 are required for H2A phosphorylation at serine 95 at *GI*. **(A)** The representative phenotype of Col-0, *mlk3-1*, *gi-1*, and *mlk3-1 gi-1* plants under a LD photoperiod is shown. **(B)** The total leaf number of Col-0, *mlk3-1*, *gi-1*, and *mlk3-1 gi-1* plants under a LD photoperiod. Values shown are mean ± SD of total leaves; At least 30 plants were scored for each line. Asterisks indicate *P* < 0.05 and ns indicates no significance by *t*-test. **(C)** The representative phenotype of Col-0, *mlk3-1mlk4-2*, *gi-1*, and *mlk3-1mlk4-2 gi-1* plants under a LD photoperiod. **(D)** The total leaf number of Col-0, *mlk3-1mlk4-2*, *gi-1*, and *mlk3-1mlk4-2 gi-1* plants under a LD photoperiod. Values shown are mean ± SD of total leaves; At least 25 plants were scored for each line. Asterisks indicate *P* < 0.01 and ns indicates no significance by *t*-test. **(E,F)** The transcript levels of *GI* were examined in *mlk3-1*, *mlk4-2* and *mlk3-1 mlk4-2* mutants **(E)**, and in *mlk3-1 mlk4-2, mlk1-3 mlk3-1 mlk4-2*, and *mlk2-3 mlk3-1 mlk4-2* plants **(F)**. The white bar indicates the light periods, and the black bar indicates the dark period. ZT, Zeitgeber time. Experiments were repeated at least three times, and the representative experiments shown indicate the mean ± SE, *n* = 3 replicates. **(G)** Gene structure of *GI*, indicating exons (boxes), introns (lines). The primers used for ChIP-PCR are marked with lines. **(H,I)** The amounts of H2AS95ph at different regions of *GI* were tested in the *mlk3-1*, *mlk4-2*, *mlk3-1 mlk4-2*, *mlk1-3 mlk3-1 mlk4-2*, and *mlk2-3 mlk3-1 mlk4-2* plants **(H)**. Ubiquitin 10 was used as internal control **(I)**. Experiments were repeated at least three times, and the representative experiments shown indicate the mean ± SE, *n* = 3 replicates.

To investigate the relationship between *MLK3* and *GI*, we crossed *gi-1* into *mlk3-1* and generated the *mlk3-1 gi-1* double mutant. The flowering time of *mlk3-1 gi-1* is similar to *gi-1* (Figures 6A,B and Supplementary Table 1). To confirm these, we generated *mlk3-1 mlk4-2 gi-1* triple mutant and found that the flowering time of *mlk3-1 mlk4-2 gi-1* is similar to that of *gi-1* under LD (Figures 6C,D and Supplementary Table 1). These results suggested that *MLK3* and *MLK4* are redundantly involved in *GI*-dependent flowering time pathway.

We then examined the transcription levels of *GI*. The decreased *GI* transcripts were further reduced in *mlk3 mlk4* plants (Figure 6E and Supplementary Figure 6A). However, mutations of *MLK1* or *MLK2* in *mlk3 mlk4* double mutants could not further reduce the attenuated *GI* transcripts in *mlk3 mlk4* plants (Figure 6F). These results are consistent with the flowering time phenotype of *mlk3 mlk4*, *mlk1 mlk3 mlk4*, and *mlk2 mlk3 mlk4* mutants.

We investigated the distribution of H2AS95ph by chromatin immunoprecipitation (ChIP), followed by the quantitative PCR measurement of DNA enrichment. The H2AS95ph was highly enriched at the promoter and the gene body at *GI* in wild type. The H2AS95ph enrichment was reduced in *mlk3* and *mlk4* single mutant, and further reduced in *mlk3 mlk4* double mutant (Figures 6G–I and Supplementary Figure 6B). We then examined the H2AS95ph levels in triple mutants and found that the H2AS95ph levels in *mlk134* and *mlk234* triple mutants are similar to those in *mlk3 mlk4* double mutant (Figures 6H,I), suggesting that MLK3 and MLK4 primarily deposited H2AS95ph at *GI*. These observations indicated that the transcription levels of *GI* are positively associated with the levels of H2AS95ph.

## Discussion

In this study, we revealed that MLKs redundantly participated in flowering time and H2AS95 phosphorylation. Loss of *MLK3* function displayed late-flowering phenotype under LD, but not SD. Mutations of *MLK3* enhanced the late-flowering phenotype of *mlk4*, but not *mlk1 mlk2* double mutant. Loss of *MLK1* or *MLK2* functions in *mlk3 mlk4* double mutant did not enhance the late-flowering phenotype of *mlk3 mlk4*. The transcript levels of *GI* were reduced in *mlk3 mlk4* double mutant, but not further attenuated in *mlk1 mlk3 mlk4* and *mlk2 mlk3 mlk4* triple mutants. Accordingly, the H2AS95ph levels of *GI* is severely reduced in *mlk3 mlk4* double mutant, but not further decreased in *mlk1mlk3 mlk4* and *mlk2 mlk3 mlk4* triple mutants. These results suggested that *MLK3* and *MLK4* are the major factors in flowering time and H2AS95 phosphorylation.

Among *MLK*s, *MLK1*, *MLK2*, and *MLK4* are redundant in leaf sharp and height development. Loss of *MLK4* function did not show any growth defects, whereas *mlk1 mlk2* double mutant only exhibited semidwarf with short hypocotyls (Wang et al., 2015; Su et al., 2017; Zheng et al., 2018). The growth defects including short petiole, and sharp and curly leaf were observed in *mlk1 mlk2 mlk4* triple mutant, but not other triple mutants such as *mlk1 mlk2 mlk3*, *mlk1 mlk3 mlk4*, and *mlk2 mlk3 mlk4*, suggesting that *MLK*s might evolve the divergent functions, and *MLK4* might be a key factor in flowering time and development factor. The previous study showed that loss of *MLK3* function resulted in early flowering (Kang et al., 2020). However, our observation showed that *mlk3* plants displayed the late-flowering phenotype under LD, but not SD. In addition, loss of *MLK3* function enhanced the late-flowering phenotype of *mlk4* mutants, but not *mlk1 mlk2* double mutant. MLK3 interacted with CCA1, and loss of function *MLK3* reversed the early-flowering phenotype of *cca1*. The inductions of *GI* in *cca1* were reversed in *cca1 mlk3* mutants, suggesting that *MLK3* is required for *GI* transcripts modulated by *CCA1*.

The *in vitro* and *in vivo* assays showed that MLK3 also contained kinase activity for the phosphorylation of H2AS95ph. H2AS95ph levels were not reduced in *mlk3* and *mlk4* single mutant, but reduced in *mlk3 mlk4* double mutant. Further study showed that H2AS95ph levels in *mlk1 mlk3 mlk4* and *mlk2 mlk3 mlk4* triple mutant are similar to that in *mlk3 mlk4* double mutant. The flowering time phenotype of *mlk3 mlk4* double mutant is similar to that of *mlk1 mlk3 mlk4* and *mlk2 mlk3 mlk4* triple mutant. MUT9p could phosphorylate H2A and H3, and H3 at Threonine 3 is the best substrate in Chlamydomonas (Casas-Mollano et al., 2008). In our study, H3T3ph levels were not reduced in *mlk1 mlk2 mlk3*, *mlk1 mlk2 mlk4*, *mlk1 mlk3 mlk4*, and *mlk2 mlk3 mlk4* triple mutant, suggesting that MLKs, unlike MUT9p, are primarily involved in H2AS95 phosphorylation.

Flowering at appropriate time determines whether plant successfully generate seeds in next generation; thus, the plant has evolutes the complicated system to regulate the flowering time. The histone modifications, including histone methylation, histone acetylation, and histone variants were observed at *FLC*, and these modifications play pivotal roles in autonomous and vernalization flowering time pathway in *Arabidopsis* (He and Amasino, 2005; Berry and Dean, 2015; Whittaker and Dean, 2017). The histone trimethylation of H3 lysine 27 was induced and histone trimethylation of H3 lysine 36 was reduced with prolonged cold treatment (Yang et al., 2014), suggesting that the histone methylations dynamically change in response to environmental change. Although the temperature and photoperiod are mostly common environment, little is known about how histone modification changes in response to photoperiod. Our study showed that defects in MLKs reduced H2AS95ph levels and transcription levels of*GI*, which, in turn, decreased the transcription levels of *CO* and *FT*. CCA1, MLK4, and YAF9a form a protein complex and promote H2A.Z deposition at *GI* (Su et al., 2017). The correlation between H2AS95ph levels and transcription levels at *GI* was at least partially due to the attenuated H2A.Z deposition. Together, our study indicated that H2AS95ph levels of *GI* are closely linked with flowering time and transcription levels of*GI*, and H2AS95 phosphorylation might be an essential activation marker in flowering time.

## Materials and Methods

### Plant Materials

The *Arabidopsis thaliana* ecotype Col-0 was grown at 22°C under a LD photoperiod in a 16-h-light/8-h-dark cycle and light intensity of 140 μmol m^–2^ s^–1^ or a SD photoperiod with 8-h-light/16-h dark. The mutant strains obtained from the SALK collection were as follows: *mlk1-2*, Salkseq_132455; *mlk1-3*, SALK_039903; *mlk2-2*, SALK_149222; *mlk2-3*, SALK_064333; *mlk3-1*, SALK_017102; *mlk3-2*, SAIL_1151_E03; *mlk4-2*, SALK_201615c; *mlk4-3*, SAILseq_317_D02.1; *cca1-22*, SALKseq_120169; *gi-1*, CS3123; and *co-9*, CS870084.

### Plasmid Constructs

The plasmids were constructed with the DNA primers and protocols described in Supplementary Data Set 1. All cloned DNAs were confirmed by DNA sequencing.

### Yeast-Two Hybrid

The yeast two-hybrid assay was performed according to the manufacturer’s protocol (Clontech; user manual 630489). Briefly, the *Saccharomyces cerevisiae* strain Y190 was transformed with the bait constructs pGBKT-MLK3 and pGADT7-CCA1. The yeast was scored for protein interaction based on their ability to grow on synthetic-defined medium lacking Trp, Leu, His, and adenine. The primers used to generate the constructs are shown in Supplementary Data Set 1.

### Phosphorylation Reaction *in vitro*

The phosphorylation reaction assay was performed as described (Su et al., 2017). Briefly, 2–3 μg purified protein was incubated with 2.5 μCi γ^–32^ P ATP in a reaction buffer (50 mM Tris–HCl, pH 7.4, 10 mM MgCl2, 50 mM NaCl, 1 mM DTT, 2 mMEDTA, and 50 mM ATP) at 30°C for 1 h. The reaction products were separated by SDS-PAGE and autoradiographed with Storage Phosphor System.

### Protein Pull-Down Assays, Co-IP, and Immunoblot Assays

For the pull-down assays, 3 μg bait and prey proteins were incubated overnight at 4°C. The beads were washed with a solution containing 20 mM Tris (pH 7.4), 150 mM NaCl, and 0.05% Tween 20 separated on a sodium dodecylsulfate-polyacrylamide gel electrophoresis (SDS-PAGE) gel and analyzed by immunoblotting using an anti-GST antibody (GenScript, Nanjing, China; A00866-100, lot: 18A001413) or an anti-His antibody (Abmart, Shanghai, China; M30111M, lot: 293871). Co-IP was performed as described previously (Su et al., 2017). Briefly, 1 × 10^6^ protoplasts were lysed with PEN-140 buffer (140 mM NaCl, 2.7 mM KCl, 25 mM Na2HPO4, 1.5 mM KH2PO4, 0.01 mM EDTA and 0.05% CA-630). The supernatant was precipitated with anti-FLAG (Sigma-Aldrich; H8592, lot: SLBV3799) antibodies, followed by washes with PEN-400 buffer (400 mM NaCl, 2.7 mM KCl, 25 mM Na2HPO4, 1.5 mM KH2PO4, 0.01 mM EDTA and 0.05% CA-630). The samples were analyzed by immunoblotting using anti-HA (Roche; 11867423001, lot: 13500600) and anti-FLAG antibodies.

### Real-Time qPCR Analysis

Total RNA was isolated from 7-day seedlings grown under a LD photoperiod. RT-PCR analysis was performed with a CFX real-time PCR instrument (Bio-Rad) and SYBR Green mixture (Roche). The relative expression of the genes was quantified with the 2^–ΔΔ*CT*^ calculation using *Ubiquitin 10* as the reference housekeeping gene for the expression analyses. The enrichment of DNA at specific genes was quantified with the 2^–ΔΔ*CT*^ calculation using *Ubiquitin 10* as the reference housekeeping gene for ChIP assays. The gene-specific primers are shown in Supplementary Data Set 1.

### Chromatin Immunoprecipitation Assay

Chromatin immunoprecipitation assay was performed as described previously (Su et al., 2017). Briefly, 7-day seedlings grown under a LD photoperiod were fixed with formaldehyde and quenched in glycine. The grounds were extracted with buffer I (0.4 M sucrose, 10 mM Tris (pH 8.0), 5 mM β-mercaptoethanol, 0.1 mM phenylmethylsulfonylfluoride (PMSF), and protease inhibitor cocktail), buffer II (0.25 M sucrose, 10 mM Tris (pH 8.0), 10 mM MgCl2, 1% Triton X-100, 5 mM β-mercaptoethanol, 0.1 mM PMSF and protease inhibitor cocktail), and buffer III (1.7 M sucrose, 10 mM Tris (pH 8.0), 10 mM MgCl2, 1% Triton X-100, 5 mM β-mercaptoethanol, 0.1 mM PMSF, and protease inhibitor cocktail). The specific antibodies anti-H2AS95ph were generated as described previously (Su et al., 2017), or control IgG serum, was added to the precleared supernatants for an overnight incubation at 4°C. The immunoprecipitated sample was extracted and analyzed by RT-PCR with the gene-specific primers shown in Supplementary Data Set 1.

### Accession Numbers

Sequence data from this article can be found in the GenBank/EMBL data libraries under the following accession numbers: *MLK1* (At5g18190), *MLK2* (At3g03940), *MLK3* (At2g25760), and *MLK4* (At3g13670).

## Data Availability Statement

The original contributions presented in the study are included in the article/Supplementary Material, further inquiries can be directed to the corresponding author/s.

## Author Contributions

YD and TH conceived the study and designed the experiments. HeZ performed the MLK3 kinase activity experiments, western blot, and ChIP-PCR assays. HaZ performed the MLK1 and MLK2 kinase activity experiments, and generated the *mlk1 mlk2* and *mlk1 mlk2 co* mutants. TH prepared the materials with the help of other authors and performed most of the experiments. YD wrote the manuscript. All authors contributed to the article and approved the submitted version.

## Conflict of Interest

The authors declare that the research was conducted in the absence of any commercial or financial relationships that could be construed as a potential conflict of interest.

## Publisher’s Note

All claims expressed in this article are solely those of the authors and do not necessarily represent those of their affiliated organizations, or those of the publisher, the editors and the reviewers. Any product that may be evaluated in this article, or claim that may be made by its manufacturer, is not guaranteed or endorsed by the publisher.
