# Supplementary material for: Phosphorylation of Histone H2A at Serine 95 Is Essential for Flowering Time and Development in Arabidopsis
Source: Front Plant Sci. 2021 Nov 23;12:761008. doi: 10.3389/fpls.2021.761008 (PMC8650089; doi:10.3389/fpls.2021.761008)
Supplement: Supplementary file 1 [file Data_Sheet_1.PDF]

Supplemental Figure 1

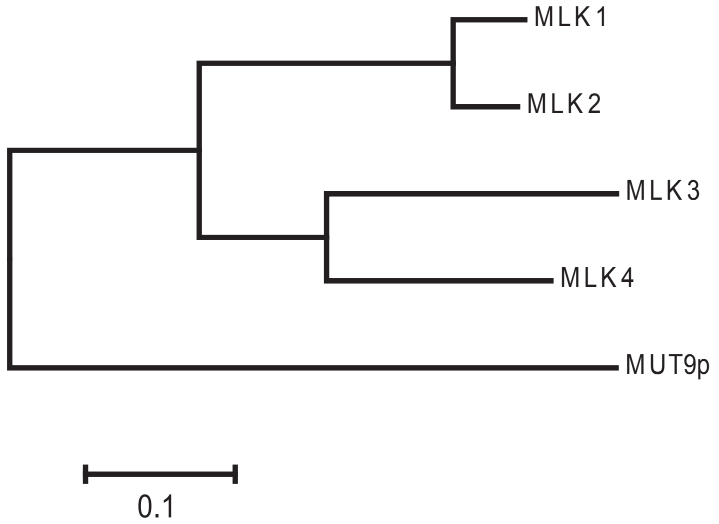

Supplemental Figure 1. Phylogenetic analysis of MUT9p in *Chlamydomonas* and MLK family proteins in *Arabidopsis*.

(A) MUT9p in *Chlamydomonas*, and MLK1, MLK2, MLK3 and MLK4 in *Arabidopsis* were aligned with ClustalW. The relationships of the sequences were examined with MEGA5. The evolutional scar bar was indicated in bottom.

Supplemental Figure 2

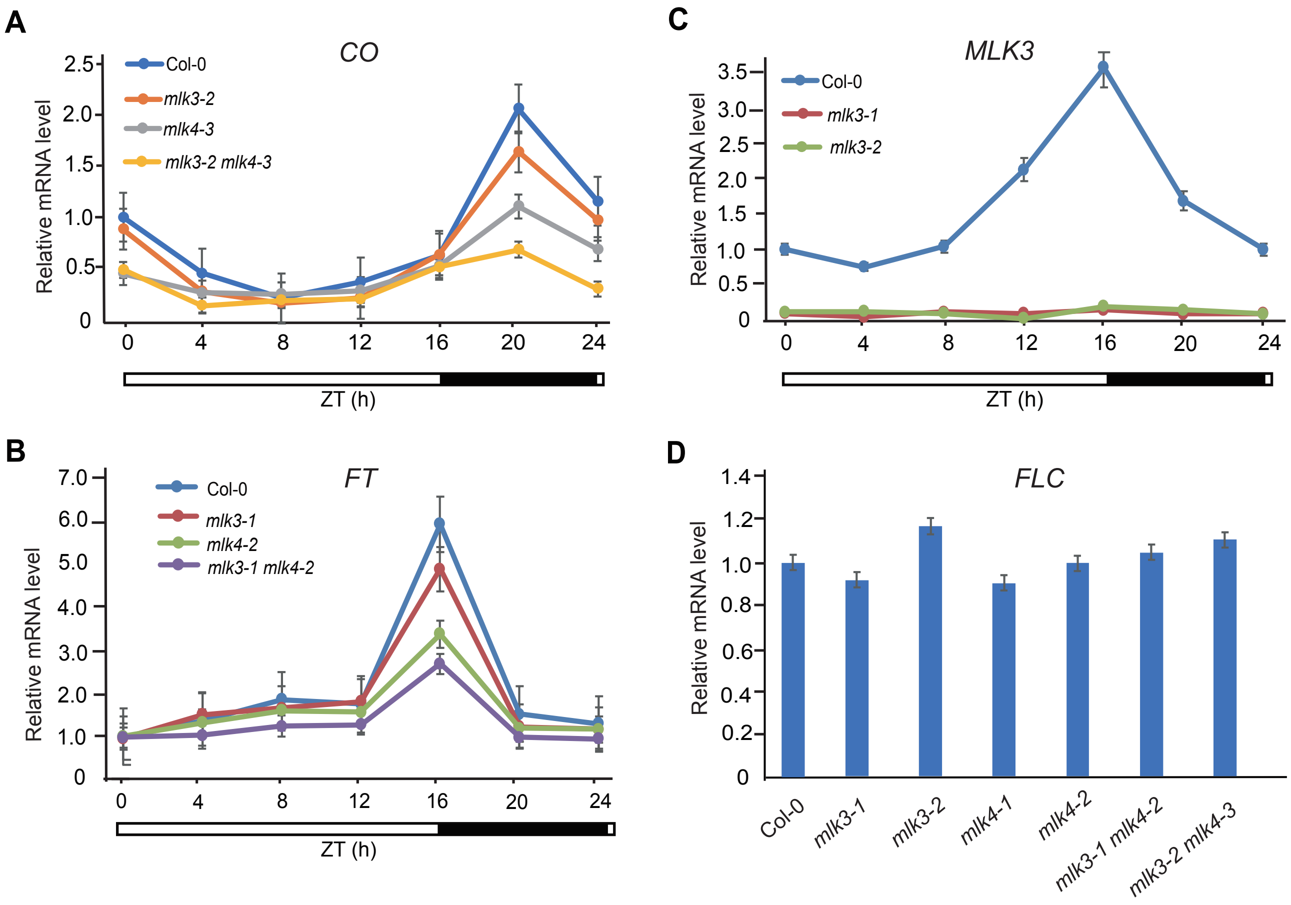

Supplemental Figure 2. The transcript levels of *CO, MLK3, FT,* and *FLC.*

The transcript levels of *CO* (A), *FT* (B)*, MLK3* (C)*,* and *FLC* (D) were examined in *mlk3*, *mlk4*, and *mlk3 mlk4* double mutants. The white bar indicates the light periods, and the black bar indicates the dark period. ZT, Zeitgeber time. Experiments were repeated at least three times, and the representative experiments shown indicate the mean ±SE, n =3 replicates.

Supplemental Figure 3

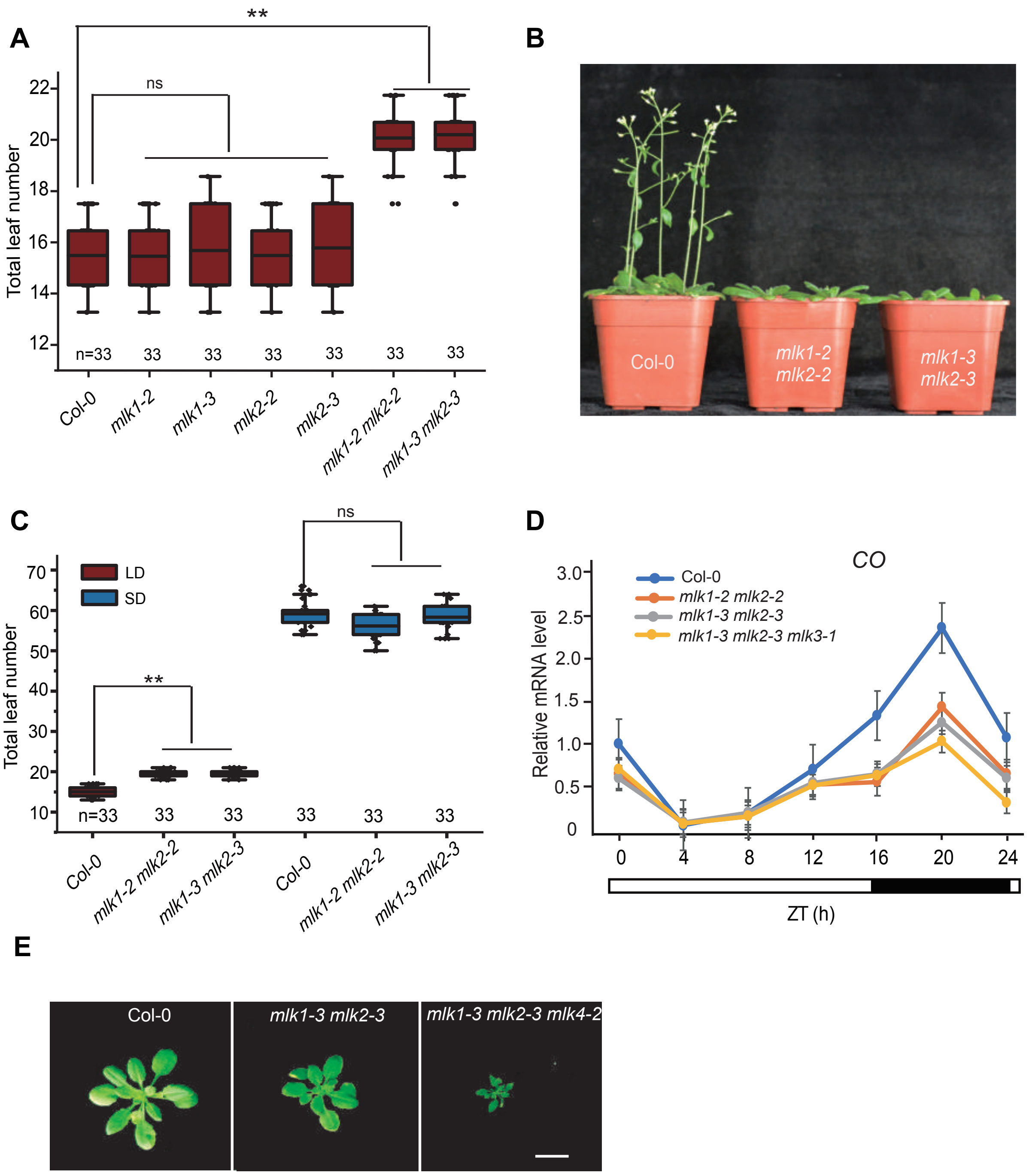

Supplemental Figure 3. The phenotype of *mlk1-3 mlk2-3*, and *mlk1-3 mlk2-3 mlk4-2*.

(A) The total leaf number of Col-0, *mlk1-2*, *mlk1-3*, *mlk2-2*, *mlk2-3*, *mlk1-2 mlk2-2*, and *mlk1-3 mlk2-3* plants under a LD and photoperiod. Values shown are mean ±SD of total leaves; 33 plants were scored for each line. Asterisks indicate P < 0.01 by t-test.

(B) The representative phenotype of Col-0, *mlk1-2 mlk2-2*, and *mlk1-3 mlk2-3* plants under a LD photoperiod.

(C) The total leaf number of Col-0, *mlk1-2 mlk2-2*, and *mlk1-3 mlk2-3* plants under a LD and photoperiod and SD photoperiod. Values shown are mean ±SD of total leaves; 33 plants were scored for each line. Asterisks indicate P < 0.01 and ns indicates no significance by t-test.

(D) The transcript levels of *CO* were examined in Col-0, *mlk1-2 mlk2-2*, *mlk1-3 mlk2-3*, and *mlk1-3 mlk2-3 mlk3-1* plants. The white bar indicates the light periods, and the black bar indicates the dark period. ZT, Zeitgeber time. Experiments were repeated at least three times, and the representative experiments shown indicate the mean ±SE, n =3 replicates.

(E) The 3-week old Col-0, *mlk1-3 mlk2-3*, and *mlk1-3 mlk2-3 mlk4-2* plants was shown. Bar=2 cm

Supplemental Figure 4

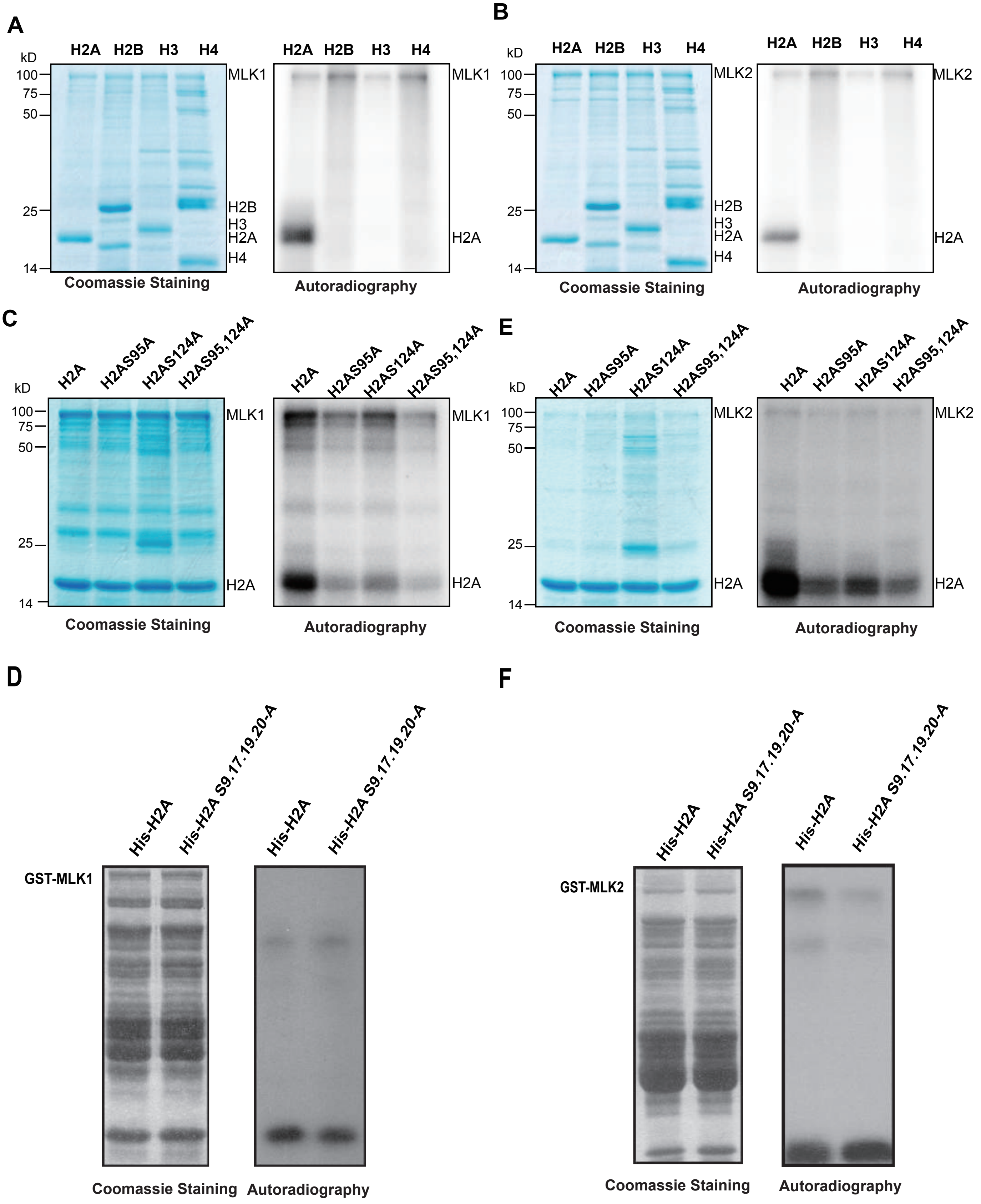

Supplemental Figure 4. MLK1 and MLK2 phosphorylated H2A

(A) and (B) MLK1 and MLK2 phosphorylated H2A. The ability of MLK1 (A) and MLK2 (B) to phosphorylate H2A, H2B, H3, and H4 was assessed. The MLK1, MLK2, H2A, H2B, H3, and H4 were marked.

(C) (D) (E), and (F) The activity and specificity of the MLK1 (C) and (D), and MLK2 kinase (E) and (F) were assessed using different substrates. Wild-type H2A and H2A containing serine-to-alanine substitutions were usedas various residues.

Supplemental Figure 5

A

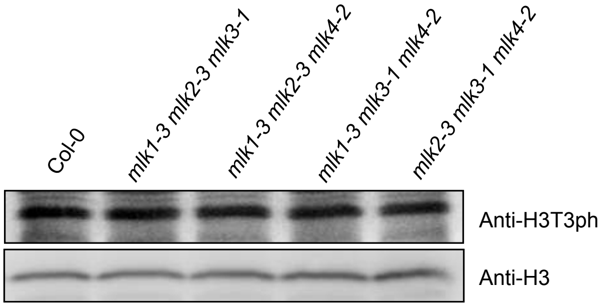

Supplemental Figure 5. The global levels of H3T3 phosphorylation.

(A) The global levels of H3T3 phosphorylation were examined in *mlk1 mlk2 mlk3, mlk1 mlk2 mlk4, mlk1 mlk3 mlk4,* and *mlk2 mlk3 mlk4* triple mutants.

Supplemental Figure 6

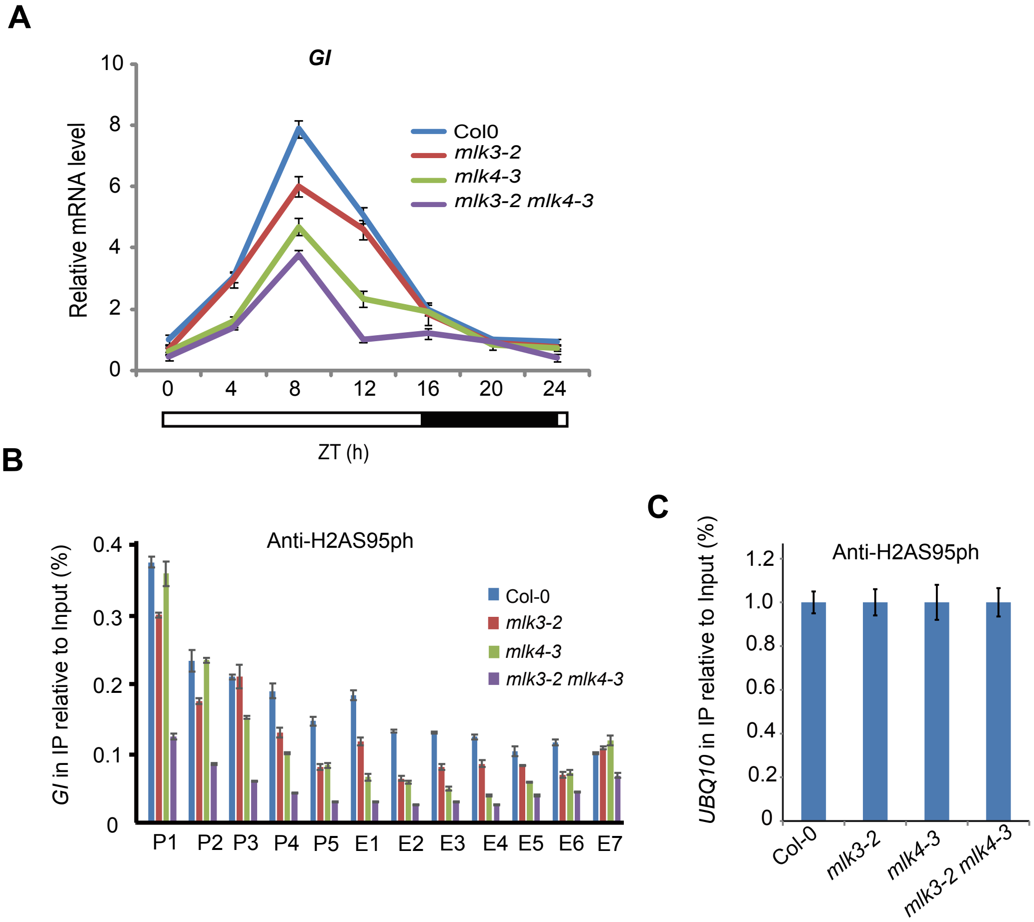

Supplemental Figure 6. The transcription levels and H2AS95ph levels in *mlk3-2 mlk4-3* plants.

(A) The transcript levels of *GI* were examined in wild type, *mlk3-2*, *mlk4-3*, and *mlk3-2 mlk4-3* plants. The white bar indicates the light periods, and the black bar indicates the dark period. ZT, Zeitgeber time. Experiments were repeated at least three times, and the representative experiments shown indicate the mean ±SE, n =3 replicates.

(B) and (C) The amounts of H2AS95ph at different regions of *GI* were tested in the *mlk3-2*, *mlk4-3*, and *mlk3-2 mlk4-3* mutants (B). *Ubiquitin 10* was used as internal control (C). Experiments were repeated at least three times, and the representative experiments shown indicate the mean ±SE, n =3 replicates.
