# Supplementary material for: Phosphorylation of Histone H2A at Serine 95 Is Essential for Flowering Time and Development in Arabidopsis
Source: Front Plant Sci. 2021 Nov 23;12:761008. doi: 10.3389/fpls.2021.761008 (PMC8650089; doi:10.3389/fpls.2021.761008)
Supplement: Supplementary file 2 [file Data_Sheet_2.PDF]

Supplemental Data Set 1. Plasmids and primers

pGBKT7-MLK3

The full length MLK3 cDNA was amplified from an Arabidopsis first strand cDNA pool using forward (5'- GCCATGGAGGGAATTCATGCCTGAGCTGCGTAGCAACGCAC-3') and reverse (5'- CAGCTTCGATGGATCCTCATGACACAGTTCGACCATAACAA-3') primers, and clonedinto pGBKT7 with EcoRI/BamHI cohesive ends.

pGBKT7-MLK4

The full length MLK4 cDNA was amplified from an Arabidopsis first strand cDNA pool using forward (5'-CCGGAATTCATGCCGGAGCTTCGCCGTGGAGTCCGCC-3') and reverse (5'-CGCGGATCCAACAGTTTTTACTAATAAGTCCCA-3') primers, and cloned into pGBKT7 with EcoRI/BamHI cohesive ends.

pGADT7-CCA1

The full length CCA1 cDNA was amplified from an Arabidopsis first strand cDNA pool using forward (5'-CGCGGATCCTGATGGAGACAAATTCGTCTGGAGA-3') and reverse (5'-CCGCTCGAGCTCATGTGGAAGCTTGAGTTTCCAA-3') primers, and cloned into pGADT7 with BamHI/XhoI cohesive ends.

GST-MLK1

The full length MLK1 cDNA was amplified from an Arabidopsis first strand cDNA pool using forward (5'-CGCGGATCCTTGTATGGAGTAAGAATGCCAGAGT-3') and reverse(5'-ACGCGTCGACTCTACATACATACGAACACGCATC-3') primers, and cloned into pGEX-6P-1 with BamHI/ SalI cohesive ends.

GST-MLK2

The full length MLK2 cDNA was amplified from an Arabidopsis first strand cDNA pool using forward (5'-CGCGGATCCAGAATGCCAGAGTTAAGAAGTG-3') and reverse (5'-ACGCGTCGACGTGAGAGAGGTTGAAACAGTAT-3') primers, and cloned into pGEX-6p-1 with BamHI/SalI cohesive ends

GST-MLK3

The full length MLK3 cDNA was amplified from an Arabidopsis first strand cDNA pool using forward (5'-TTCCAGGGCTGGGATCCATGCCTGAGCTGCGTAGCAACGCAC-3') and reverse(5'-CTCGAACCCGGGAATTCTCATGACACAGTTCGACCATAACAA-3') primers, and cloned into pGEX-6P-1 with EcoRI/ SalI cohesive ends.

GST-MLK4

The full length MLK4 cDNA was amplified from an Arabidopsis first strand cDNA pool using forward (5'-ATCGGATCCATGCCGGAGCTTCGCCGTGGAGTCCGCC-3') and reverse (5'-GAATTCTCAAGATACAGTTCGGCCATAGCTTAA -3') primers, and cloned into pGEX-6p-1 with BamHI/EcoRI cohesive ends.

GST-CCA1

The full length CCA1 cDNA was amplified from an Arabidopsis first strand cDNA pool using forward (5'-ATCGGATCCTGGAGACAAATTCGTCTGGAGAAG-3') and reverse (5'-GAATTCACTCATGTGGAAGCTTGAGTTTCCAAC-3') primers, and cloned into pGEX-6p-1 with BamHI/EcoRI cohesive ends.

His-MLK3

The full length MLK3 cDNA was amplified from an Arabidopsis first strand cDNA pool using forward (5'-ACGCGTCGACATGCCTGAGCTGCGTAGCAACGCAC-3') and reverse(5'-ATAAGAATGCGGCCGCTTCATGACACAGTTCGACCATAACAA-3') primers, and cloned into pET30a with SalI /NotI cohesive ends.

His-MLK4

The full length MLK4 cDNA was amplified from an Arabidopsis first strand cDNA pool using forward (5'- ACGCGTCGACATGCCGGAGCTTCGCCGTGGAGTCC -3') and reverse (5'- ATAAGAATGCGGCCGCTCAAGATACAGTTCGGCCATAGCTT -3') primers, and cloned into pET30a with SalI /NotI cohesive ends.

YFP^N-MLK3

The full length MLK3 cDNA was amplified from an Arabidopsis first strand cDNA pool using forward (5'- GAGAACAACTCTAGAATGCCTGAGCTGCGTAGCAACGCAC-3')

and reverse (5’- GACAGTAATGGATCCTCATGACACAGTTCGACCATAACAA-3’) primers, and cloned into pUC-SPYNE with BamHI/XhoI cohesive ends.

YFP^N-MLK4

The full length MLK4 cDNA was amplified from an Arabidopsis first strand cDNA pool using forward (5’-ATCGGATCCATGCCGGAGCTTCGCCGTGGAGTCCGCC-3’) and reverse (5’-AGTCTCGAGAGATACAGTTCGGCCATAGCTTAAC-3’) primers, and cloned into pUC-SPYNE with BamHI/XhoI cohesive ends.

YFP^C-CCA1

The full length CCA1 cDNA was amplified from an Arabidopsis first strand cDNA pool using forward (5’-ACGCGTCGACATGGAGACAAATTCGTCTGGAGAAG-3’) and reverse (5’-CGGGGTACCTGTGGAAGCTTGAGTTTCCAAC-3’) primers, and cloned into pUC-SPYCE with SalI/KpnI cohesive ends.

PUC19-HA-MLK3

The full length MLK3 cDNA was amplified from an Arabidopsis first strand cDNA pool using forward (5’- ATTTACGAAGTCGACATGCCTGAGCTGCGTAGCAACGCAC-3’) and reverse (5’- GTGGTCCTCGTCGACTCATGACACAGTTCGACCATAACAA-3’) primers, and cloned into pUC19-HA with SpeI cohesive ends.

PUC19-HA-MLK4

The full length MLK4 cDNA was amplified from an Arabidopsis first strand cDNA pool using forward (5’-GGACTAGTATGCCGGAGCTTCGCCGTGGAGTCC-3’) and reverse (5’-GGACTAGTAGATACAGTTCGGCCATAGCTTAAC-3’) primers, and cloned into pUC19-HA with SpeI cohesive ends.

PUC19-FLAG-CCA1

The full length CCA1 cDNA was amplified from an Arabidopsis first strand cDNA pool using forward (5’-ACGCGTCGACATGGAGACAAATTCGTCTGGAGAAG-3’) and reverse (5’-CGGGTCGACTGTGGAAGCTTGAGTTTCCAAC-3’) primers, and cloned into pUC19-FLAG with SalI cohesive ends.

Supplemental Table 2. Primers

Genotyping primers

mlk1-2 (AT5g18190 SALK_026482)
forward primer (5’-AAAGCTCTTGTTGTCACCCTG-3’)
reverse primer (5’-TTTTGGTCCACCTTACGAGTG-3’)

mlk1-3 (AT5g18190 SALKseq_132455)
forward primer (5’-TGTTCCATAATCCAGTCCTGC-3’)
reverse primer (5’-TGGAAAGATTCACACTCAGGC-3’)

mlk2-2 (AT3g03940 SALK_149222)
forward primer (5’-CGAGACGCCTTGACGAGCAGCCTA-3’)
reverse primer (5’-GGCTGTTCATCCTCTTCCAAAT-3’)

mlk2-3 (AT3g03940 SALK_064333)
forward primer (5’-TGAGTGGTGGCAGTGATAGGATT-3’)
reverse primer (5’-GGCTGTTCATCCTCTTCCAAAT-3’)

mlk3-1 (AT2g25760 SALK_017102)
forward primer (5’- GAGTCTCCTGTGTCTCATCGG-3’)
reverse primer (5’- TCGTGTTTGTCTGGTGTGAAG-3’)

mlk3-2 (AT2g25760 SAIL_1151_E03)
forward primer (5’- GGTCTGAACAAGAGCACTTGC-3’)
reverse primer (5’- ACCAAATCACCTTCTGTGCAC-3’)

mlk4-2 (AT3g13670 Salk_201615c)
forward primer (5’-GGAGATAACAAGTCATTCCTT-3’)
reverse primer (5’-ACTGCTGTGTATACTGCGTAC-3’)

mlk4-3 (AT3g13670 SAILseq_317_D02.1)
forward primer (5’-CAAATTAGTCGCAAGTCACGG -3’)
reverse primer (5’-CACAGCAGTAAACGCAGACTG-3’)

cca1-22 (AT2G46830 SALKseq_120169)
forward primer (5’-ATCCGATTCCAAGAAATCCTG-3’)
reverse primer (5’-TGGAAAACGGTAATGAGCAAG-3’)

gi-1 (AT1G22770)
forward primer (5’-ATTATATAACCTCATAGATGTTCAC-3’)
reverse primer (5’-AAAGATTGAGCTACTTCGTCATTTA-3’)

co-9 (AT5G15840)
forward primer (5’-AAGCTGTTGTGACACATGCTG-3’)
reverse primer (5’-CCCCTTCTTTCAGATACCAGC-3’)

qPCR primers

UBQ10 (AT4G05320)
forward primer (5’-AGGATGGCAGAACTCTTGCT-3’)
reverse primer (5’-TCCCAGTCAACGTCTTAACG-3’)

GI (AT1G22770)
forward primer (5’-TCTTGCGAATTTCCTCACAG-3’)
reverse primer (5’-GATTTCAGGAGCAGCAATCA-3’)

CO (AT5G15840)
forward primer (5’-GGCTCCTCAGGGACTCACTA-3’)
reverse primer (5’-GGGTGTGAAGCTGTTGTGAC-3’)

FT (AT1G65480)
forward primer (5’-TCCATTGGTTGGTGACTGAT-3’)
reverse primer (5’-GCCAAGCTGTCGAAACAATA-3’)

ChIP PCR primers

GI (AT1G22770)

Region P1
forward primer (5’-ACGTAAGGTTTGAGCCCATC-3’)
reverse primer (5’-ACGATTCGTGATTGTGTGGT-3’)

Region P2
forward primer (5’-GACAGAAGTGGACCACACAATC-3’)
reverse primer (5’-CGTTGATCACTCGCCAAATA-3’)

Region P3
forward primer (5’-CGAGTGATCAACGTCCACA-3’)
reverse primer (5’-AAATGGGCCTCACACATCTT-3’)

Region P4
forward primer (5’-CTAGCTGCCCGAAATCTCTT-3’)
reverse primer (5’-GGAGCTAAGTTCAGATGCTACG-3’)

Region P5
forward primer (5’-GCAAAGGCAAGGAAATATCG-3’)
reverse primer (5’-GTGGTGGCCTTAGGAGAGAG-3’)

Region E1
forward primer (5’-TCTGAGAGATGGATCGATGG-3’)
reverse primer (5’-GCAATGTCATCTGGGAATTG-3’)

Region E2
forward primer (5’-AGCATGGTCATGCAGTCATT-3’)
reverse primer (5’-CCTCACTACTTGGGCAAACTAA-3’)

Region E3
forward primer (5’-GACTCATTACAACCGTCCCA-3’)
reverse primer (5’-TCCTTTCATGCTGTGTTGGT-3’)

Region E4
forward primer (5’-TCCTCAACTGATGCCTTCAA-3’)
reverse primer (5’-GCTGTCAGCGTAGCAGTCTC-3’)

Region E5
forward primer (5’-TTGAGGCGAATCAACCTGTA-3’)
reverse primer (5’-ACAGGCAAGAGCACAAACAG-3’)

Region E6
forward primer (5’-TCGCCAAAGATGATGAAGAA-3’)
reverse primer (5’-CAGTGACTCAGGCCTGTCAT-3’)

Region E7
forward primer (5’-GCCAACAACAGAGAAGAACG-3’)
reverse primer (5’-GGCATAGTTGTGGAGAGCAA-3’)
