# Supplementary material for: Phosphorylation of Histone H2A at Serine 95 Is Essential for Flowering Time and Development in Arabidopsis
Source: Front Plant Sci. 2021 Nov 23;12:761008. doi: 10.3389/fpls.2021.761008 (PMC8650089; doi:10.3389/fpls.2021.761008)
Supplement: Supplementary file 3 [file Data_Sheet_3.PDF]

Supplemental Table 1. Primary Leaf Number at Bolting of *mlk3*, *mlk4*, *cca1*, *co*, *gi* Mutants in LD and SD

	LD		SD	
Mutant	Rosette Leaves	Cauline Leaves	Rosette Leaves	Rauline Leaves
Col-0	12.03±0.91	3.05±0.55	48.79±3.33	9.78±0.97
mlk3-1	13.14±0.78	3.41±0.5	50.92±1.99	7.61±1.07
mlk3-2	13.39±0.71	3.21±0.42	50.64±3.23	8.42±1.55
co-9	25.45±1.87	5.20±0.41	50.14±2.23	8.09±1.03
mlk3-1/co-9	25.30±1.49	6.50±0.82	51.13±3.14	7.20±0.92
mlk4-2	19.43±1.43	4.95±0.69	49.63±2.42	8.88±1.02
mlk3-1/mlk4-2	23.95±1.10	5.25±0.63	49.18±3.03	8.75±0.68
mlk1-3/mlk2-3/mlk3-1	15.25±0.72	3.13±0.72	47.31±2.79	8.81±0.75
mlk1-3/mlk3-1/mlk4-2	27.93±1.63	5.93±1.07	38.24±3.39	9.22±0.81
mlk2-3/mlk3-1/mlk4-2	27.73±1.38	6.33±0.61	34.00±3.52	8.67±0.77
mlk1-3/mlk2-3/mlk4-2	19.47±1.55	5.60±0.63	34.69±2.63	7.55±0.85
mlk1-3/mlk2-3	17.15±1.31	3.20±0,56	49.86±2.77	8.75±0.79
mlk1-3/mlk2-3/co-9	25.85±2.43	5.21±0.86	49.85±1.89	8.15±0.56
cca1-21	9.60±0.68	3.28±0.45		
mlk3-1/cca1-21	13.62±0.49	3.14±0.36		
gi-1	29.86±1.45	6.38±0.50		
mlk3-1/gi-1	29.33±1.98	7.14±0.72		
mlk3-1/mlk4-2/gi-1	30.00±2.21	6.75±1.01		

Values shown are mean number± SD of rosette and cauline leaves; at least 30 plants were scored for each line.
